# Physical activity guidelines and cardiovascular risk in children: a cross sectional analysis to determine whether 60 minutes is enough

**DOI:** 10.1186/s12889-016-2708-7

**Published:** 2016-01-22

**Authors:** L. M. Füssenich, L. M. Boddy, D. J. Green, L. E. F. Graves, L. Foweather, R. M. Dagger, N. McWhannell, J. Henaghan, N. D. Ridgers, G. Stratton, N. D. Hopkins

**Affiliations:** 1 Department of Physiology, Radboud University Nijmegen Medical Centre, Nijmegen, Netherlands; 2 Research Institute for Sport and Exercise Science, Liverpool John Moore’s University, Tom Reilly Building, Byrom Street, Liverpool, L3 2ET UK; 3 School of Sports Science, Exercise and Health, The University of Western Australia, Crawley, Perth, WA 6009 Australia; 4 School of Health Sciences, Liverpool Hope University, Liverpool, L16 9JD UK; 5 Department of Sport and Exercise Science, University of Chester, Chester, CH1 4BJ UK; 6 Centre for Physical Activity and Nutrition (C-PAN) Research, Deakin University, 221 Burwood Highway, Burwood, VIC 3175 Australia; 7 Applied Sports Technology Exercise and Medicine Research Centre (A-STEM), Swansea University, Swansea, SA2 8PP UK

**Keywords:** Physical activity guidelines, Moderate/vigorous physical activity, Clustered cardiovascular risk

## Abstract

**Background:**

Physical activity reduces cardiovascular mortality and morbidity. The World Health Organisation (WHO) recommends children engage in 60 min daily moderate-to-vigorous physical activity (MVPA). The effect of compliance with this recommendation on childhood cardiovascular risk has not been empirically tested. To evaluate whether achieving recommendations results in reduced composite-cardiovascular risk score (CCVR) in children, and to examine if vigorous PA (VPA) has independent risk-reduction effects.

**Methods:**

PA was measured using accelerometry in 182 children (9–11 years). Subjects were grouped according to achievement of 60 min daily MVPA (active) or not (inactive). CCVR was calculated (sum of z-scores: DXA body fat %, blood pressure, VO_2_peak, flow mediated dilation, left ventricular diastolic function; CVR score ≥1SD indicated ‘higher risk’). The cohort was further split into quintiles for VPA and odds ratios (OR) calculated for each quintile.

**Results:**

Active children (92 (53 boys)) undertook more MVPA (38 ± 11 min, *P* < 0.001), had greater VO_2_peak (4.5 ± 0.8 ml/kg/min *P* < 0.001), and lower fat % (3.9 ± 1.1 %, *P* < 0.001) than inactive. No difference were observed between active and inactive for CCVR or OR (*P* > 0.05). CCVR in the lowest VPA quintile was significantly greater than the highest quintile (3.9 ± 0.6, *P* < 0.05), and the OR was 4.7 times higher.

**Conclusion:**

Achievement of current guidelines has positive effects on body composition and cardiorespiratory fitness, but not CCVR. Vigorous physical activity appears to have beneficial effects on CVD risk, independent of moderate PA, implying a more prescriptive approach may be needed for future VPA guidelines.

## Background

Physical activity (PA) predicts cardiovascular disease (CVD) morbidity and mortality [1] and prevents and/or reduces traditional and emerging cardiovascular (CV) risk factors in healthy and high risk children [2, 3]. The American College of Sports Medicine originally published PA recommendations for young people in 1988, albeit based on their adult recommendations [4]. These guidelines and the supporting evidence base have been re-evaluated numerous times in an attempt to account for advances in PA and CVD risk factor measurement techniques [5, 6]. Most recently, The World Health Organization (WHO) updated their paediatric PA recommendations [7] to reflect findings from a review by Janssen and LeBlanc [2] and the European Youth Heart Study [8], which suggested that previous guidelines underestimated the activity necessary to reduce CVD risk in young people. The WHO guidelines now suggest children aged 5–17 years accumulate 60 min of moderate-to-vigorous physical activity (MVPA) daily, in addition to everyday physical activities, and that vigorous intensity physical activity (VPA) should be incorporated at least three times per week.

Whilst the updated guidelines address some of the limitations of previous versions [3] and advocate more PA than previously, numerous limitations remain. No study has empirically tested guidelines by comparing CVD risk in children who do achieve them to those who do not, whilst both Andersen et al. and Strong et al. [3, 9] provide comprehensive reviews on the dose response relationship, and make important recommendations for childhood PA, they do not test the validity of current recommendations using empirical data. Secondly, current guidelines are based on self-report PA, which has numerous limitations, and do not include novel CVD risk markers as outcome measures. Andersen et al. [3] addressed these limitations via inclusion of objective PA data, and novel inflammatory markers. However, endothelial and diastolic dysfunction are yet to be included in such analyses despite the crucial role each plays in the development of CVDs [10], their strong prognostic capacity in predicting CV events [11, 12], and clear associations with PA [13–15]. Finally, evidence suggests VPA may have potent effects on CVD risk [16], yet, WHO recommendations on VPA specifically, remain vague. With these limitations in mind, we sought to evaluate whether adherence to current WHO recommendations equates to a reduction in CVD risk in children. Secondly, we aimed to examine if, and in what quantity, VPA provides additional CVD risk benefits beyond moderate PA (MPA).

## Methods

### Subjects

Data were generated by the REACH Year 6 and the Active City of Liverpool, Active Schools and SportsLinx (A-CLASS) studies (Liverpool, UK). Fourteen schools from areas of social deprivation (IMD >40) participated across the studies; all 9–11 year old children were invited to participate. Following parental consent and medical screening, 233 children (10.8 ± 0.6 years; 100 boys) were studied. All were healthy and not suffering from cardiovascular/metabolic conditions. Informed written parent/guardian consent and child assent were obtained. Ethical approval was obtained from Liverpool John Moores University Ethics Committee. All data collection methods were standardised between the studies unless otherwise stated.

### Experimental design

Initially, children visited the laboratory to complete measurements, including anthropometric tests and dual-energy X-ray absorptiometry (DXA), VO_2_peak testing, vascular endothelial function and echocardiography. Assessments were made in a quiet temperature-controlled room at the same time of day, following a morning fast and avoidance of strenuous PA for 24 h. Subsequently, PA was assessed via accelerometry over 7 consecutive days.

### Measurements and post-test analysis

#### Anthropometry and body composition

Body mass (kg), stature and sitting height (cm) were measured using standard methods. Somatic maturity was estimated by calculating time to peak height velocity (TPHV) using gender specific regression equations [17, 18].

A DXA scan (QDR discovery A, Hologic, MA) was completed according to standardized manufacturer procedures. Participants were scanned in the supine position while wearing t-shirt and shorts.

#### Vascular function

Following 20 min supine rest, brachial artery diameter, blood flow and shear rate were assessed via high-resolution ultrasonography (Acuson, Aspen, Penn and Terason, T3000, Aloka, UK) prior to, and following, 5 min forearm ischaemia. Methods were identical to those previously described [14] in accordance with best practice guidelines [19].

#### Left ventricular diastolic function

Following 10 min of quiet rest in the left lateral decubitus position. Left ventricular diastolic function was assessed via echocardiography (Mylab30CV system, ESAOTE, Italy). All system settings including gain, filter, PRF, sector size and depth were adjusted to optimise image quality. Mitral inflow was assessed from the apical four-chamber via a 2 mm sample volume at the tips of the mitral leaflets, parallel with flow, peak early (E) and late/atrial (A) velocities were obtained and E/A ratio reported.

#### Cardiorespiratory fitness

During each protocol VO_2_ and VCO_2_ were measured breath-breath via an online gas analysis system (Jaeger Oxycon Pro, Viasys Health Care, Warwick, UK). Heart rate (HR) was monitored continuously (Polar, Kempele, Finland).

##### A-CLASS study method

Peak oxygen uptake (VO_2_peak) was determined during a discontinuous treadmill exercise test which involved walking and running until volitional exhaustion. The test consisted of 3 min stages, followed by a 30-s rest interval. Peak VO_2_ was accepted as the highest 15-s averaged oxygen uptake achieved during the test with a respiratory exchange ratio ≥1.05 and/or HR ≥195 beats.min^−1^.

##### REACH study method

To account for differences in biological age and limb length, treadmill speeds were individually calibrated by anchoring speeds to set Froude (Fr) numbers as described previously [20]. A continuous protocol was used unitl volitional exhaustion occurred. Peak VO_2_ was defined as the highest 15-s average oxygen uptake achieved with a respiratory exchange ratio >1.05 and/or HR > 199 beats/min^−1^.

#### Physical activity

Physical activity was objectively measured for 7 consecutive days using a hip mounted uni-axial accelerometer (GT1M model, ActiGraph, FL, USA) set to 5 s epochs. Children wore the accelerometer during all waking hours, except during water-based activities. Consecutive zero counts >20 min were removed from analysis as non-wear. Minimum wear time for inclusion in data analysis was 9 h/day for any 3 days of the week [21]. Accelerometer data reduction was performed using ActiLife v 6.1.4 (ActiGraph, LLC, 2010–2012). The Evenson cut-points [22] were used to define PA and sedentary intensity thresholds [23]. Total time spent in each PA/SB threshold for each valid day was divided by the total number of valid days. Subjects were then split into groups; those who achieved a daily average of 60 min MVPA (active) and those who did not (inactive [24]).

### Statistical analysis

All statistical analysis were performed using SPSS (18.0, Chicago, Illinois) software. Statistical significance was set at *P* < 0.05. Variables were tested for normality when grouped by sex and PA level using the Kolmogorov-Smirnov test. DBP and E/A ratio data were normalised by log transformation and FMD using square root transformation. All analyses were performed at the cohort level initially; additional analyses were then performed by gender.

Gender specific standardized z-scores for percentage body fat, VO_2_peak, FMD, systolic blood pressure and E/A ratio were calculated and inverted where necessary, z-scores were summed to create a composite CVD risk score (CCVR). Pearson’s correlation analysis was used to assess relationships between z scores (Table 1). Children with CCVR ≥1 SD were defined as ‘higher risk’ [8]. Differences in individual CVD risk factors and CCVR between the active and inactive group, boys and girls were assessed using an independent *t*-test, or analysis of covariance (ANCOVA), with TPHV as a covariate (continuous variables), or a chi-square test (discrete variables).

**Table 1:** Correlations between z scores used to calculate CCVD risk score

Z score	VO2max (ml/kg/min)		Fat % (DXA)		SBP (mmHg)		FMD %		E/A ratio	
	*r*	*p*	*r*	*p*	*r*	*p*	*r*	*p*	*r*	*p*
VO2max (ml/kg/min)	1		−0.691	0.000	−0.163	0.036	0.025	0.743	0.173	0.027
Fat % (DXA)	−0.691	0.000	1		0.239	0.001	−0.056	0.448	−0.230	0.002
SBP (mmHg)	−0.163	0.036	0.239	0.001	1		−0.112	0.134	−0.068	0.370
FMD %	0.025	0.743	−0.056	0.448	−0.112	0.134	1		0.072	0.340
E/A ratio	0.173	0.027	−0.230	0.002	−0.068	0.370	0.072	0.340	1	

*Fat %* percentage of body fat, *DXA* dual-energy X-ray absorptiometry, *SBP* systolic blood pressure, *VO*_*2*_ *max* peak oxygen uptake, *FMD* flow mediated dilation, *E/A ratio* ratio between passive and active filling of the left ventricle (cm/s)

The cohort was split into quintiles according to VPA. Differences in individual CVD risk factors and CCVR across quintiles were assessed using ANCOVA with MPA, sedentary behaviour and TPHV as covariates, or using a chi-square test (discrete variables). Logistic regression was then used to obtain odds ratios for each group (Boys/Girls, Active/Inactive and all VPA quintiles).

## Results

### Subject characteristics

Of the 233 children initially recruited, 182 children met criteria for accelerometer wear time coupled with adequate vascular measurements. Those who did not meet criteria were removed from analyses, there were no differences between included and excluded children across measured variables. Baseline characteristics are presented in Table 2. Boys had significantly higher VO_2_peak and DBP, whilst girls were significantly closer to PHV and had higher percentage body fat. Additionally, boys engaged in significantly more MVPA and VPA than girls (Table 2).

**Table 2:** Descriptive statistics

	Group	Girls (105)	Boys (77)
Age (years)	10.8 (0.6)	10.7 (0.6)	10.8 (0.6)
Height (cm)	145.0 (8.0)	145.4 (7.8)	144.6 (8.3)
Body mass (kg)	41.7 (10.6)	42.3 (10.2)	40.9 (11.0)
Maturity offset (TPHV)	−2.51 (1.23)	−2.30 (1.44)	−2.79 (.79)
BMI (kg/m^2^)	19.6 (3.5)	19.8 (3.3)	19.3 (3.7)
Fat % (DXA)	27.7 (6.7)	29.4 (5.7)	25.2 (7.2)^a^
SBP (mmHg)	106 (11)	105 (11)	108 (12)
DBP (mmHg)	63 (6)	62 (5)	64 (6)^a^
VO_2_max (ml/kg/min)	46.0 (6.9)	43.5 (6.1)	49.6 (6.4)^a^
FMD %	8.9 (4.1)	8.9 (4.1)	8.9 (4.3)
E/A ratio	2.1 (0.5)	2.1 (0.4)	2.1 (0.5)
CCVD risk	−0.04 (2.94)	0.12 (2.98)	−0.27 (2.89)
MVPA (min)	64 (25)	56 (20)	75 (27)^a^
VPA (min)	25 (13)	22 (12)	30 (13)^a^
Sedentary (min)	605 (148)	608 (80)	600 (209)
MPA/VPA ratio	1.7 (0.6)	1.8 (0.7)	1.7 (0.6)

*TPHV* time to peak height velocity, *BMI* body mass index, *Fat %* percentage of body fat, *DXA* dual-energy X-ray absorptiometry, *SBP* systolic blood pressure, *DPB* diastolic blood pressure, *VO*_*2*_ *max* peak oxygen uptake, *FMD* flow mediated dilation, *E/A ratio* ratio between passive and active filling of the left ventricle (cm/s), *CCVD risk* composite cardio vascular disease risk score as sum of z-scores, *MVPA* moderate to vigorous physical activity, *VPA* vigorous physical activity. All data expressed as mean (SD). ^a^Significant difference between boys and girls <0.05

### Active vs. inactive analysis

Inactive children had significantly higher percentage body fat and lower VO_2_peak compared to active children. No other significant differences were found between the two groups (Table 3). The odds for being ‘higher risk’ were 1.9 (95 % CI: 0.8–4.3) times higher in the inactive group than the active group (*p* = 0.126).

**Table 3:** Cardiovascular risk factors in the active and inactive

	Group			Girls			Boys		
	Inactive (*n* = 90)	Active (*n* = 92)	*p*	Inactive (*n* = 66)	Active (*n* = 39)	*p*	Inactive (*n* = 24)	Active (*n* = 53)	*p*
Fat %	29.6 (5.9)	25.7(7.0)	>0.001	29.2 (5.9)	29.8 (5.4)	0.90	30.7 (5.8)	22.7 (6.5)	>0.001
SBP	106 (12)	106 (12)	0.89	106 (10)	104 (11)	0.58	108 (11)	108 (12)	0.97
Baseline artery diameter (mm)	3.1 (0.3)	3.1 (0.5)	0.73	3.0 (0.3)	3.0 (0.4)	0.67	3.2 (0.4)	3.1 (0.5)	0.90
FMD%	9.1 (4.1)	8.7 (4.2)	0.45	9.1 (4.1)	8.6 (3.9)	0.65	9.2 (4.0)	8.8 (4.4)	0.44
VO2max	43.70 (6.1)	48.3 (6.9)	>0.001	43.1 (6.3)	44.2 (5.7)	0.16	45.56 (5.2)	51.2 (6.2)	0.01
E/A ratio	2.2 (0.5)	2.1 (0.4)	0.78	2.2 (0.5)	2.2 (0.4)	0.36	2.1 (0.6)	2.2 (0.4)	0.64
CCVD risk	0.36 (2.95)	−0.43 (2.90)	0.40	0.09 (2.93)	0.19 (3.12)	0.87	1.22 (2.92)	−0.85 (2.70)	0.01
At risk (%)	23	13	0.35	18	18	0.99	37	10	0.03
OR CCVD risk	1.9	1.0	0.13	1.0	1.0	0.99	5.1	1.0	0.02
MVPA (min)	45 (10)	83 (21)	>0.001	45 (10)	77 (17)	>0.001	46 (9)	88 (22)	>0.001
VPA (min)	16 (6)	33 (14)	>0.001	16 (6)	30 (15)	>0.001	17 (5)	36 (12)	>0.001
MPA/VPA ratio	1.9 (0.7)	1.6 (0.6)	>0.001	1.9 (0.7)	1.7 (0.7)	0.18	1.9 (0.6)	1.6 (0.5)	>0.01
Sedentary (min)	625 (80)	585 (192)	0.03	629 (84)	573 (59)	>0.001	613(68)	594 (248)	0.53

Inactive = those who did not achieve the recommended 60 min MVPA per day. Active = those who achieved the recommended 60 min MVPA per day

*Fat %* percentage of body fat, *SBP* systolic blood pressure, *BD* baseline diameter of brachial artery in mm, *FMD* flow mediated dilation, *VO*_*2*_ *max* peak oxygen uptake, *E/A ratio* ratio between passive and active filling of the left ventricle (cm/s), *CCVD risk* clustered cardio vascular disease risk score as sum of z-scores, *At risk* percentage of children with more than 1SD in the CCVD risk score, *OR CCVD risk* odds ratio for being at risk compared to the active group, *MVPA* moderate to vigorous physical activity in minutes/day, *VPA* vigorous physical activity in minutes/day, *sedentary* sedentary behaviour in hours/day. All data expressed as mean (SD)

### Gender analysis

Boys were more active than girls (*p* < 0.001, Table 3) and active boys engaged in more MVPA than active girls (11 min/day, *p* = 0.004). Active boys had significantly lower percentage body fat and CCVR, and higher VO_2_peak than inactive boys (Table 3). Thirty seven percent of inactive boys were classed as ‘at risk’ compared to 10 % of the active boys (*p* = 0.028), the odds of being at risk were 5.1 times higher in the inactive boys than the active boys (95 % CI: 1.4 – 19.1, *p* = 0.015). Active girls engaged in significantly less sedentary behaviour compared to inactive girls (*p* < 0.001), no further differences were found.

### Vigorous physical activity

**Fig. 1:**
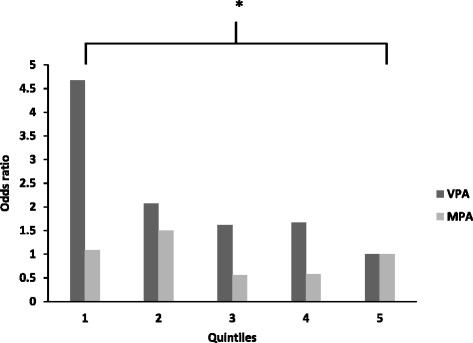
Odds ratios for being at risk by quintiles of physical activity. *Significant difference between VPA quintile 1 and 5, *P* < 0.05. No significant differences across MPA quintiles. VPA = vigorous physical activity, MPA = moderate physical activity

Comparisons were made between the highest VPA quintile (Q5) and all others (Table 4). CCVR was significantly elevated in Q1. Q1, Q2 and Q4 had significantly higher percentage body fat. VO_2_peak was significantly lower in Q1 and Q2. The OR was significantly higher in Q1 than Q5 (OR 4.7, *p* < 0.05, Table 4; Fig. 1). When quintiles were examined by gender, no significant differences were found between quintiles (difference between Q1 and Q5: 6.1 (95 % CI: 0.6 – 59.5, *p* < 0.05) and 7.4 (95 % CI: 0.7 – 80.0, *p* < 0.05), in girls and boys respectively).

**Table 4:** Cardiovascular risk factors in quintiles of vigorous physical activity

Whole cohort	Number	VPA	MPA	Sedentary	MPA/VPA ratio	CCVD risk	At risk (%)	OR	Fat %	FMD%	SBP	VO2max	E/A ratio
1	36	11 (2)^a^	27 (10)^a^	632 (91)	2.5 (0.8)^a^	1.31 (3.26)^a^	34.5 %^a^	4.7^a^	31.9 (1.2)^a^	8.4 (3.8)	106 (12)	42.3 (6.7)^a^	2.0 (0.3)
2	37	17 (2)^a^	34 (8)^a^	613 (64)	2.0 (0.5)^a^	0.23 (2.93)	20.7 %	2.1	29.3 (1.0)^a^	9.5 (4.9)	104 (12)	43.6 (5.3)^a^	2.2 (0.4)
3	36	22 (2)^a^	36 (7)^a^	603 (69)	1.6 (0.3)	−0.40 (2.81)	17.2 %	1.6	27.1 (1.0)	8.3 (3.7)	105 (9)	46.7 (6.7)	2.3 (0.6)
4	37	29 (2)^a^	43 (11)^a^	587 (56)	1.5 (0.4)	−0.06 (2.58)	17.2 %	1.7	27.5 (1.0)^a^	9.1 (4.2)	108 (12)	46.9 (6.9)	2.0 (0.3)
5	36	45 (14)	56 (13)	587 (303)	1.3 (0.3)	−1.22 (2.71)	10.3 %	1	22.4 (1.3)	9.3 (4.0)	106 (11)	51.0 (5.8)	2.1 (0.5)
Boys													
1	15	13 (2)^a^	30 (11)^a^	613 (72)	2.3 (0.8)^a^	1.11 (3.38)	36.4 %	7.4	30.0 (7.0)	8.8 (4.5)	105 (13)	47.5 (6.1)	1.9 (0.3)
2	16	20 (2)^a^	39 (6)^a^	590 (73)	1.9 (0.3)^a^	0.25 (2.63)	18.8 %	3.0	27.4 (6.5)	9.0 (4.0)	106 (11)	47.1 (7.2)	2.3 (0.6)
3	15	28 (3)^a^	42 (11)^a^	586 (22)	1.5 (0.3)	−0.20 (2.68)	15.4 %	2.4	24.7 (7.2)	7.3 (3.7)	111 (13)	50.8 (5.7)	2.1 (0.4)
4	16	36 (2)^a^	51 (9)^a^	675 (42)	1.4 (0.2)	−0.72 (3.04)	14.3 %	2.2	23.7 (6.9)	10.0 (5.3)	108 (10)	49.7 (5.5)	2.2 (0.5)
5	15	50 (9)	63 (14)	530 (48)	1.3 (0.2)	−1.56 (2.56)	7.1 %	1	20.2 (5.4)	9.6 (3.7)	110 (11)	53.0 (6.1)	2.2 (0.4)
Girls													
1	21	10 (2)^a^	25 (8)^a^	652 (99)^a^	2.5 (0.8)^a^	0.51 (3.16)	27.8 %	6.2	29.6 (6.8)	8.9 (4.0)	107 (11)	42.3 (5.9)	2.1 (0.3)
2	21	15 (1)^a^	33 (9)^a^	625 (75)	2.2 (0.6)^a^	0.98 (3.73)	30.0 %	6.9	32.2 (6.3)^a^	8.3 (3.9)	103 (14)	40.7 (5.9)	2.2 (0.5)
3	21	20 (1)^a^	31 (6)^a^	601 (57)	1.6 (0.3)	−0.69 (2.25)	5.6 %	0.9	28.9 (5.3)	10.1 (4.6)	104 (8)	44.0 (5.4)	2.3 (0.5)
4	21	26 (2)^a^	39 (8)	605 (69)	1.5 (0.3)	0.43 (2.71)	20.0 %	4.0	29.3 (4.6)	7.7 (3.8)	106 (9)	45.6 (5.5)	2.0 (0.5)
5	21	37 (18)	45 (13)	559 (69)	1.3 (0.7)	−0.79 (2.62)	5.9 %	1	27.2 (4.8)	9.6 (3.9)	104 (11)	45.3 (6.9)	2.1 (0.3)

*VPA* vigorous physical activity (min/day), *MPA* moderate physical activity (min/day), *CCVD risk* clustered cardiovascular disease risk score as sum of z-scores, *At risk* % of children with more than 1SD in the CCVD risk score, *OR CCVD risk* odds ratio for being at risk compared to the active group, *Fat %* percentage of body fat, *FMD%* flow mediated dilation, *SBP* systolic blood pressure, *VO2max* peak oxygen uptake, *E/A ratio* ratio between passive and active filling of the left ventricle (cm/s). All data expressed as mean (SD). CCRD risk score consists of Fat%, FMD%, SBP, VO2Max, E/A ratio. ^a^Significant difference compared to most active quintile <0.05

## Discussion

To our knowledge, this is the first study which used a composite risk score which included novel pre-clinical markers of CVD risk to empirically evaluate the effects of adherence to the current WHO PA guidelines for children, and provide novel information regarding recommendations for VPA. Our findings from the whole cohort indicate no difference in CCVR between active and inactive asymptomatic 9–11 year old children, implying current guidelines may underestimate the PA necessary to reduce CCVR. Although achieving WHO PA recommendations did have beneficial effects on VO_2_peak and adiposity. Furthermore, VPA appeared to provide health benefits in addition to those of MPA, suggesting it necessary to incorporate a more specific recommendation regarding the VPA.

Active boys have 1/5 of the risk compared to inactive boys (Table 3), whereas no differences were found between active and inactive girls. We observed a significant difference in MVPA between active boys and active girls, (11 ± 5 min, *p* = 0.004; Table 3) and active girls had a similar risk factor profile to inactive girls and inactive boys. This suggests that gender differences in risk factors may not be resultant of gender *per se*, but are possibly determined by differences in PA levels [2, 3, 25, 26]. Although active girls engaged in daily MVPA in excess 60 min, this appears insufficient to confer the CVD risk benefits afforded by the active boys. Our data suggest 77 min MVPA was insufficient for CCVR risk reduction, whilst 88 min MVPA resulted in a reduction, supporting previous conclusions from Andersen et al. [8] that PA guidelines should be higher than the current 60 min MVPA/day. Furthermore, our data raise the question of whether children’s PA guidelines should be gender specific; little is known about gender specific dose responses to PA, further investigation therefore appears to be warranted.

In agreement with adult literature, which demonstrate that VPA is a more meaningful predictor of cardiometabolic risk than MPA [27], we provide evidence that VPA may afford additional CVD risk reduction in children. CCVR ORs were ~5 times higher in Q1 compared to Q5 (*p* < 0.05), whilst no differences were observed across MPA quintiles (Fig. 1). The largest reduction in risk was evident between Q1 and Q2 (VPA 11 ± 2 vs 17 ± 2 min, respectively, *p* > 0.05; Table 4) and although this risk reduction is non-significant in this small study, we suggest that a reduction in odds ratio of ~50 % is a clinically meaningful finding. On this basis, 17 min of VPA per day (Q2), equating to around 2 h VPA/week is needed to reduce CVD risk in pre-pubertal children.

In contrast to percent body fat and VO_2_ peak, there was no significant difference between active and inactive groups or across VPA quintiles for E/A ratio or FMD. Whilst the inclusion of FMD and E/A ratio in analysis provides novel information, these surrogate markers do not appear to enhance the predictive power of the CCVR model in this cohort, One possible interpretation of this finding is that diastolic and endothelial function are not modulated by PA, however a wealth of previous findings contradict this hypothesis [13, 14, 28]. A more plausible hypothesis relates to the dose of activity the inactive children are exposed to; 45 min MVPA/day, including 17 min of VPA, whilst they fall short of WHO guidelines for MVPA, this level of VPA may be adequate to prevent deterioration of endothelial and diastolic function. These data lend support to our recommendations above that 17 min VPA/day is cardioprotective, and previous findings from our group which imply that VPA is more important for endothelial function than other PA intensities [14]. Further research is needed to confirm or refute the role of VPA in the modulation of these variables.

This study advances knowledge of the complex relationship between PA and CVD risk in children, as it investigates, for the first time, the utility of adding novel CVD surrogates, to a composite score of ‘pre-clinical’ markers to estimate CVD risk. As CVD risk factors tend to cluster in sedentary and obese individuals, stronger associations between CVD risk and PA may be observed when a composite CVD risk score is generated. Additionally, as we measured PA levels objectively we are confident of greater measurement precision than the self-report PA data from which the current WHO guidelines are derived, nonetheless given the sporadic nature of children’s PA patterns, it is plausible that using an epoch length of 5 s may result in an underestimation of VPA. Furthermore, a lack of parity in PA measurement techniques and accelerometer cut points used between this and other studies makes comparison and interpretation difficult. Various confounders including diet, smoking status and socioeconomic status were not accounted for in our analyses as we lack the data to do so. Finally, although our findings lend support to the previous recommendations that 60 min daily MVPA may not be enough for children of this age [8], given the relatively small sample size and limited number of children that achieved guidelines, findings should be interpreted with caution.

## Conclusions

In our study of asymptomatic 9–11 year old children, there were no differences between CCVR of children who undertook 60 min MVPA per day in accordance with WHO recommendations, and those who did not. This implies that current recommendations may be an underestimation of the PA necessary to reduce clustered CVD risk. A gender difference between the CVD risk in active and inactive children, raises the possibility that gender specific guidelines may be needed, although much work is needed to determine if these differences are a result of gender specific responses to PA or sex differences in PA level. Finally, VPA appears to provide CCVR benefits beyond those afforded by MPA, with data suggesting that 17 min VPA/day may provide clinically meaningful CVD risk reductions. Taken together these findings suggest that in order to reduce CVD risk, the current guidelines should be updated to increase the amount of MVPA recommended, and to prescribe a daily amount of VPA.

## Abbreviations

PA: physical activity
VPA: vigorous physical activity
MPA: moderate physical activity
MVPA: moderate to vigorous physical activity
CVD: cardiovascular disease
WHO: World Health Organisation
CCVR: clustered cardiovascular risk
OR: odds ratio
DXA: dual-energy X-ray absorptiometry
FMD: flow mediated dilation
TPHV: time to peak height velocity

## References

[1] Mora S, Cook N, Buring JE, Ridker PM, Lee IM (2007). Physical activity and reduced risk of cardiovascular events: Potential mediating mechanisms. Circulation.

[2] Janssen I, Leblanc AG (2010). Systematic review of the health benefits of physical activity and fitness in school-aged children and youth. Int. J. Behav. Nutr. Phys. Act..

[3] Andersen LB, Riddoch C, Kriemler S, Hills AP (2011). Physical activity and cardiovascular risk factors in children. Br J Sports Med.

[4] Medicine. ACoS (1988). Physical fitness in children and youth. Med Sci Sports Exerc.

[5] Biddle S, Sallis JF, Cavill N (1999). Young and active? Young people and health-enhancing physical activity—evidence and implications.

[6] Sallis JF, Patrick K (1994). Physical Activity Guidelines for Adolescents: Consensus Statement. Pediatr Exerc Sci.

[7] Organisation WH (2010). Global recommendations on physical activity for health.

[8] Andersen LB, Harro M, Sardinha LB, Froberg K, Ekelund U, Brage S (2006). Physical activity and clustered cardiovascular risk in children: a cross-sectional study (The European Youth Heart Study). Lancet.

[9] Strong WB, Malina RM, Blimkie CJ, Daniels SR, Dishman RK, Gutin B (2005). Evidence based physical activity for school-age youth. J Pediatr.

[10] Landmesser U, Hornig B, Drexler H (2004). Endothelial function: a critical determinant in atherosclerosis?. Circulation.

[11] Khouri SJ, Maly GT, Suh DD, Walsh TE (2004). A practical approach to the echocardiographic evaluation of diastolic function. J. Am. Soc. Echocardiogr..

[12] Yeboah J, Folsom AR, Burke GL, Johnson C, Polak JF, Post W (2009). Predictive value of brachial flow-mediated dilation for incident cardiovascular events in a population-based study: the multi-ethnic study of atherosclerosis. Circulation.

[13] Watts K, Beye P, Siafarikas A, Jones T, Davis E, Green DJ (2004). Exercise training in obese children: Effects on vascular function and body composition. J Pediatrics.

[14] Hopkins N, Stratton G, Tinken TM, McWhannell N, Ridgers ND, Graves LEF (2009). Relationships between measures of fitness, physical activity, body composition and vascular function in children. Atherosclerosis.

[15] Triposkiadis F, Ghiokas S, Skoularigis I, Kotsakis A, Giannakoulis I, Thanopoulos V (2002). Cardiac adaptation to intensive training in prepubertal swimmers. European J Clin Invest.

[16] Rakobowchuk M, McGowan CL, de Groot PC, Hartman JW, Phillips SM, Macdonald MJ (2005). Endothelial function of young healthy males following whole-body resistance training. J Appl Physiol.

[17] Sherar LB, Mirwald RL, Baxter-Jones AD, Thomis M (2005). Prediction of adult height using maturity-based cumulative height velocity curves. J Pediatr.

[18] Mirwald RL, Baxter-Jones AD, Bailey DA, Beunen GP (2002). An assessment of maturity from anthropometric measurements. Med Sci Sports Exerc.

[19] Thijssen DH, Black MA, Pyke KE, Padilla J, Atkinson G, Harris RA (2011). Assessment of flow-mediated dilation in humans: a methodological and physiological guideline. Am J Physiol Heart Circ Physiol.

[20] Hopkins N, Stratton G, Maia J, Tinken TM, Graves LE, Cable TN (2010). Heritability of arterial function, fitness, and physical activity in youth: a study of monozygotic and dizygotic twins. J Pediatr.

[21] Cain KL, Sallis JF, Conway TL, Van Dyck D, Calhoon L (2013). Using accelerometers in youth physical activity studies: a review of methods. J Phys Act Health.

[22] Evenson KR, Catellier DJ, Gill K, Ondrak KS, McMurray RG (2008). Calibration of two objective measures of physical activity for children. J Sports Sci.

[23] Trost SG, Loprinzi PD, Moore R, Pfeiffer KA (2011). Comparison of accelerometer cut points for predicting activity intensity in youth. Med Sci Sports Exerc.

[24] Sedentary Behaviour Research N (2012). Letter to the editor: standardized use of the terms “sedentary” and “sedentary behaviours”. Appl. Physiol. Nutr. Metab..

[25] Hopkins ND, Stratton G, Tinken TM, Ridgers ND, Graves LE, McWhannell N (2011). Seasonal reduction in physical activity and flow-mediated dilation in children. Med Sci Sports Exerc.

[26] Pahkala K, Heinonen OJ, Lagstrom H, Hakala P, Simell O, Viikari JS (2008). Vascular endothelial function and leisure-time physical activity in adolescents. Circulation.

[27] Janssen I, Ross R (2012). Vigorous intensity physical activity is related to the metabolic syndrome independent of the physical activity dose. Int J Epidemiol.

[28] Obert P, Mandigout S, Vinet A, N’Guyen LD, Stecken F, Courteix D (2001). Effect of aerobic training and detraining on left ventricular dimensions and diastolic function in prepubertal boys and girls. Int J Sports Med.

